# Suppression of TGF-β/Smad3 signaling pathway by *Capparis spinosa* and quercetin in a rat model of nonalcoholic steatohepatitis

**DOI:** 10.22038/IJBMS.2024.76264.16497

**Published:** 2024

**Authors:** Reza Afarin, Mahdi Hatami, Sajad Monjezi, Fatemeh Bineshfar, Akram Ahangarpour

**Affiliations:** 1 Diabetes Research Center, Health Research Institute, Ahvaz Jundishapur University of Medical Sciences, Ahvaz, Iran; 2 Student Research Committee, Ahvaz Jundishapur University of Medical Sciences, Ahvaz, Iran; 3 Medicinal Plant Research Center, Ahvaz Jundishapur University of Medical Sciences, Ahvaz, Iran

**Keywords:** Capparis spinosa, NAFLD/NASH, Quercetin, Saroglitazar, TGF-β/Smad3

## Abstract

**Objective(s):**

Liver diseases, including non-alcoholic fatty liver disease (NAFLD) and non-alcoholic steatohepatitis (NASH), pose significant global public health challenges. This study investigates the therapeutic effects of quercetin (QC), *Capparis spinosa* (CS), a QC and CS combination, and Saroglitazar (SARO) on NASH in a Wistar rat model.

**Materials and Methods:**

NASH was induced by a 42-day high-fat diet regimen in male Wistar rats. Post-induction, rats were divided into five groups receiving SARO, QC, CS, and CS+QC combination. We monitored changes in liver and body weights and evaluated the expression of genes associated with fatty acid biosynthesis (e.g., ACC and FAS), β-oxidation (e.g., CPT1, PPAR α), inflammation (e.g., TNF-α and IL-6), and fibrosis (e.g., TGF-β and COL1A), as well as protein expression levels of p-Smad2/3 and p-Smad3.

**Results:**

Treatment with QC+CS significantly decreased liver weight, body mass gain, and liver triglyceride (TG) compared to other treatments. The QC and CS combined therapy also resulted in a greater normalization of hepatic enzymatic activities, including decreases in ALT and AST levels, coupled with improvements in lipid profile indicated by decreased LDL-C and increased HDL-C concentrations, as compared to SARO and QC alone. Furthermore, this combined treatment significantly down-regulated the expression of TGF-β, TNF-α, IL-6 genes, and Smad2/3 and Smad3 protein levels.

**Conclusion:**

Our study demonstrates that an interactive effect between QC and CS can effectively reduce liver fibrosis and steatosis by inhibiting the TGF-β/Smad3 signaling pathway in a diet-induced model of nonalcoholic steatohepatitis and fibrosis in rats.

## Introduction

Non-alcoholic fatty liver disease (NAFLD) stands as a prevalent global metabolic disorder that has been on the rise, presenting its most severe manifestation, non-alcoholic steatohepatitis (NASH), which poses significant health risks (1). This complex disease is characterized by accumulation of lipid droplets in liver cells, leading to hepatic inflammation and hepatocyte damage, particularly in advanced NASH stages, with or without fibrosis (2). The progression of NAFLD and NASH is impacted by various factors, including oxidative damage, inflammation, insulin resistance, modified lipid metabolism, and the production of cytokines and adipokines (2-4). Consequently, there is an urgent need to identify effective therapeutic strategies to combat the progression of these conditions.

In the pathogenesis of NAFLD and NASH, tumor necrosis factor-alpha (TNF-α) assumes a pivotal role by intensifying inflammation and contributing to hepatic steatosis, oxidative stress, and hepatocyte apoptosis (5). Moreover, TNF activity orchestrates the production of proinflammatory cytokines, including monocyte chemoattractant protein-1 (MCP-1) and TGF-β, among others (6). TGF-β1, the predominant isoform in the liver, has been implicated in stellate cell activation and extracellular matrix protein production (7), while MCP-1, mainly secreted by hepatic stellate cells (HSCs) and macrophages, promotes hepatic lipid deposition, insulin resistance, and leukocyte migration. Additionally, interleukin-6 (IL-6) has been shown to enhance insulin resistance, liver vulnerability, hepatocyte apoptosis, and NASH progression (7).

Studies suggest that focusing on PPAR α/γ receptors, crucial components of lipid and glucose metabolism primarily located in adipose tissue, liver, and skeletal muscles, may offer a promising avenue for treating metabolic diseases (8). Dual PPAR α/γ ligands have demonstrated potential in this context, as PPAR α activation improves hyperlipidemia by promoting beta-oxidation-related gene expression, while PPAR γ increases adipogenesis and fatty acid transport gene expression, leading to adipocyte accumulation (9). Moreover, PPAR γ activation has been reported to reduce MCP-1 expression while increasing adiponectin receptors in adipose tissue (10).

*Capparis spinosa*, colloquially known as caper, is a perennial plant native to the Mediterranean region, the Middle East, and certain parts of Asia. Notably, it has a history of traditional use due to its varied pharmacological properties, encompassing anti-inflammatory, antioxidant, and hepatoprotective effects. The bioactive compounds present in *C. spinosa*, such as quercetin, rutin, catechin, and gallic acid, have been found to exert beneficial effects on NAFLD and NASH, which are characterized by hepatic steatosis, inflammation, and fibrosis. (11-13). Quercetin, a prominent flavonoid extensively present in fruits, vegetables, and herbs, showcases diverse biological functions. These encompass antioxidant, anti-inflammatory, anti-apoptotic, hepatoprotective, and cardioprotective properties (14, 15). Flavonoids found in foods have the ability to substantially reduce the degree of liver damage. Quercetin treatment ameliorates inflammation and fibrosis in nonalcoholic steatohepatitis, reduces hepatic fat accumulation, and mitigates the effects of high-fructose and high-cholesterol-induced liver steatosis (16).

This study aims to investigate the interactive effect between *C. spinosa* and quercetin in reducing diet-induced NASH and fibrosis in a rat model by suppressing the TGF-β/Smad3 signaling pathways, thereby providing new insights into potential therapeutic interventions for these metabolic disorders.

## Materials and Methods

***Providing high-fat emulsion***

The formulation for the high-fat diet employed to induce hepatic steatosis is presented in Table 1. It consisted of 79% fat, 8% carbohydrates, and 15% protein-rich whole milk powder. The high-fat diet formulation was prepared according to the guidelines provided by previous study (17, 18). This emulsion was stored at 4 ^°^C and warmed in a 40 ^°^C water bath daily before use.

***Providing chemicals***

Quercetin (QC), was procured from Sigma-Aldrich (St. Louis, USA). Saroglitazar was supplied by Cadila Healthcare Limited, Ahmedabad, India. The drugs were suspended in 0.5% sodium carboxymethyl cellulose solution (CMC) for administration.

***Preparation of plant extract and determination of yield***

*C. spinosa* (CS) samples were collected in Ahvaz, located in Khuzestan, Iran, in July 2021. The samples were taxonomically identified and stored in the Hyperlipidemia Research Center (Ahvaz, Iran). The crude extract was prepared following the methods described by Eddouks *et al*. (19). After harvesting, the CS fruit was washed with deionized water, dried at 40 ^°^C, and ground to a fine powder. Ten grams of fruit powder were mixed with 100 ml of distilled water for three hours. This mixture was heated for 10 min, followed by 15 min of cooling. Subsequently, a 0.2 mm Millipore filter was used to filter the aqueous extract (Yvelines, France). The extract was then freeze-dried and stored at -20 ^°^C until needed. The percentage of yielding extract was calculated as follows:

Yield percentage=(weight of sample extract/initial weight of sample)×100

***Experimental design and animals***

Adult male Wistar rats, weighing between 185 and 205 g, were acclimatized for 7 days at the Experimental Animal Center (Ahvaz, Iran). The animals were housed in cages under controlled environmental conditions, which included a 12-hour light-dark cycle, a room temperature of 25.5 ^°^C, and a relative humidity of 58%. This study was approved by the University Ethics Committee (IR.AJUMS.ABHC.REC.1400.065), and all procedures were performed in accordance with the laws and guidelines governing the care of research animals. Initially, 48 rats were divided into two groups: the Control (CON) group (n=8) and the HFD group (n=40). The HFD group received a high-fat emulsion (10 ml/kg)(20) daily via gavage for 7 weeks. All animals had *ad libitum* access to standard food and water throughout the duration of the experiment. They were also allowed unrestricted access to water containing 19% sucrose to simulate a more prevalent form of steatohepatitis. In the seventh week, two rats from the control group and four rats from the HFD group were randomly sacrificed to confirm the presence of NAFLD/NASH. Their livers were sent to our pathology laboratory for analysis. Upon confirming the efficacy of the model, pharmacological treatments were individually administered for six weeks, starting from the eighth week. The rats in the HFD group were divided into five groups. The first group received a high-fat emulsion (10 ml/kg) daily. The second group received a high-fat diet with 3 mg/kg of body weight of SARO (20), and The third group received a high-fat emulsion with QC at a dose of 0.8 g/kg (21) of body weight. The fourth group received a high-fat diet along with CS extract at a dose of 20 mg/kg (19) body weight, and the fifth group received a high-fat diet along with combined CS+QC at 20 mg/kg+0.8 g/kg body weight. At the end of the treatments, rats that had fasted for 15 hr were sacrificed after receiving a lethal dose of ketamine-xylazine. Blood samples were collected from the heart ventricles. The livers were removed, rinsed with cold saline, and placed on sterilized filter paper. Liver weight was immediately measured to calculate the liver index (liver weight/body weight×100). Portions of liver tissue were then aliquoted, and equal amounts were snap-frozen in liquid nitrogen at -185 ^°^C for future gene expression studies. Another liver section was preserved in 10% formalin for histological examination.

***Biochemical analysis***

Serum concentrations of hepatic enzymes, including AST and ALT, as well as the lipid profile, were assessed using a Roche 6000 auto-analyzer and corresponding test kits. Serum AST and ALT concentrations were determined using enzymatic colorimetric assays (Pars Azmun, Iran). High-density lipoprotein cholesterol (HDL-C) and low-density lipoprotein cholesterol (LDL-C) levels were measured using a Hitachi 912 auto-analyzer with homogeneous enzymatic colorimetric methods (Pars Azmun, Iran).

***Histopathological evaluations***

The liver sections underwent dehydration and were subsequently embedded in paraffin. The paraffin-embedded sections were stained with hematoxylin-eosin (HE) to facilitate histopathological assessments, specifically focusing on hepatic steatosis, inflammation, and fibrosis. The grading of steatosis was determined based on the percentage of hepatocytes involved, taking into account the macrovesicular fat content (Grade 1: 0-25%; Grade 2: 26-50%; Grade 3: 51-75%; Grade 4: 76-100%)(22). The degree of inflammation and fibrosis was explained by the mean of 10 various fields in every slide, which were grouped on a scale of 0-3 (0: normal; 1: mild; 2: moderate; 3: severe) as described by Avni *et al* . (23).

***Detection of reactive oxygen species in liver tissue***

Following the collection of liver tissues from Rats, samples were weighed and resuspended in PBS (phosphate-buffered saline, pH 7.4) to achieve a final concentration within the range of 10-50 mg tissue per milliliter. After a homogenization step performed on ice, samples were centrifuged at 10,000 g for 5 min at 4 ^°^C. The resultant supernatant was then used for subsequent ROS detection and protein concentration measurements, to ensure that data were normalized for tissue variations.

To assay ROS, the supernatant was mixed with a DCFH solution prepared as directed by the ROS detection kit provided by Cell Biolabs, Inc. (San Diego, USA). This solution, protected from light, facilitated the oxidation of DCFH to the fluorescent compound DCF within the samples, a process directly indicative of ROS presence. Fluorescence intensities were measured at excitation and emission wavelengths of 480 nm and 530 nm, respectively, with fluorescence plate reader technology. Protein concentrations were determined using the bicinchoninic acid (BCA) assay kit (Thermo Fisher Scientific, USA), favoring its compatibility with the sample matrix and its wider range of accurate detection. Bovine serum albumin solutions were prepared to create a standard curve for protein quantitation, ensuring that ROS levels measured could be expressed on a per-milligram protein basis, thus normalizing the readings for consistency and comparability. Samples, standards, and controls were processed in sets of triplicates to guarantee the reliability and reproducibility of results. All assay procedures were performed as outlined by the ROS detection kit’s protocol.

***Quantitative polymerase chain reaction (qPCR)***

The expression of relevant genes listed in Table 2 was assessed through qPCR. Following the treatments, total RNA was extracted from frozen liver tissues using an RNA extraction kit (Yekta Tajhiz Azma Company, Iran) in accordance with the manufacturer’s guidelines. Subsequently, cDNA synthesis was conducted utilizing the Yekta Tajhiz Azma cDNA kit as per the instructions, employing random hexamer and oligo-dT primers. To measure mRNA levels, real-time PCR was executed using the RealQ Plus 2x Master Mix Green “low Rox” kit (Ampliqon, Denmark) and the QuantStudio™ 3 Real-Time PCR System (ABI Applied Biosystems). The procedure initiated with a hot start, heating the PCR solution to 95 ^°^C for 15 min, followed by 40 cycles of denaturation at 95 ^°^C for 15 sec, and annealing/extension at 60 ^°^C for 1 min. The total runtime of this protocol on our system was 110 min, employing the specified primers.

***Western blot analysis***

The content of p-Smad3 and p-Smad2/3 proteins was evaluated by western blot analysis. Following a wash with phosphate-buffered saline, the liver tissue was lysed in radioimmunoprecipitation assay (RIPA) buffer containing protease inhibitors, and the protein concentration was determined using a bicinchoninic acid (BCA) assay kit (Thermo Fisher Scientific, USA). The isolated proteins were transferred onto a polyvinylidene fluoride membrane (Millipore, USA). The membranes were treated with primary antibody against phosphorylation of p-Smad3 (Ser423/425) and p-Smad2/3 (Thr8) proteins (1:1000; Cell Signaling, USA). After three washes with PBS‐Tween20, Peroxidase-conjugated goat anti-rabbit IgG secondary antibody (1:10,000; Cell Signaling, USA) was incubated with the membranes at room temperature for 1 hr. Protein bands were visualized using an electrochemiluminescence (ECL) detection kit (GE Healthcare, Chicago, IL, USA). The amount of protein was normalized to the expression level of GAPDH and semi-quantified with ImageJ software.

***Statistical analysis***

A one-way analysis of variance (ANOVA), followed by Tukey’s *post hoc* test for multiple comparisons, was performed on the data using Prism version 9.0.2. Statistical significance was defined as a *P*-value of less than 0.05.

## Results

***Alterations in body mass and liver index following treatment***

**Figure 1:**
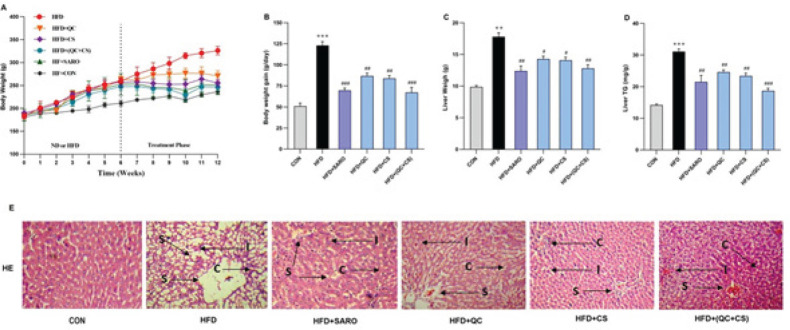
Changes in body mass and liver index, triglyceride (TG), and morphology in the Wistar rats

Figures 1A and B demonstrate that there was no significant difference in the initial body mass. Compared to the control group, rats that were administered a high-fat diet for 13 weeks exhibited considerable increases in body mass, liver weight, and liver triglycerides (TG). In all treated groups, body weight elevations were notably normalized following six weeks of treatment in comparison to the HFD group. In contrast to the HFD group, treatment with SARO, QC, CS, and QC+CS significantly decreased liver weight and liver TG (Figure 1C, D). The histopathological examination of liver tissue revealed that rats that were exclusively nourished with an HFD exhibited a significantly elevated degree of hepatic steatosis. The staining properties of hematoxylin and eosin (H&E) in sections of the liver (as depicted in Figure 1E) indicate the reverse progression of steatosis caused by HFD through intragastric administration of QS+CS.

***CS and QC combination reduces liver enzymes in the HFD model***

**Figure 2:**
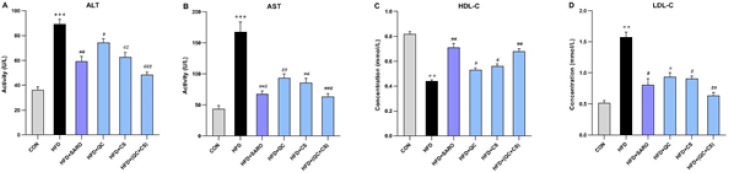
Changes in lipid profiles and liver enzymes before and after drug treatment in the Wistar rats

This study utilized ALT and AST as markers for liver damage. Rats fed the high-fat emulsion exhibited elevated ALT and AST blood levels compared to the control group (Figure 2 A, B). The combination of CS and QC ameliorated the adverse effects of the high-fat diet, reducing AST and ALT levels as well as high-density lipoprotein cholesterol (HDL-C) and low-density lipoprotein cholesterol (LDL-C) (Figure 2 C, D). The CS and QC combination proved more effective than other groups in decreasing and normalizing ALT and AST levels and lipid profiles.

***Combined CS and QC group modulates lipid-related gene expression***

**Figure 3:**
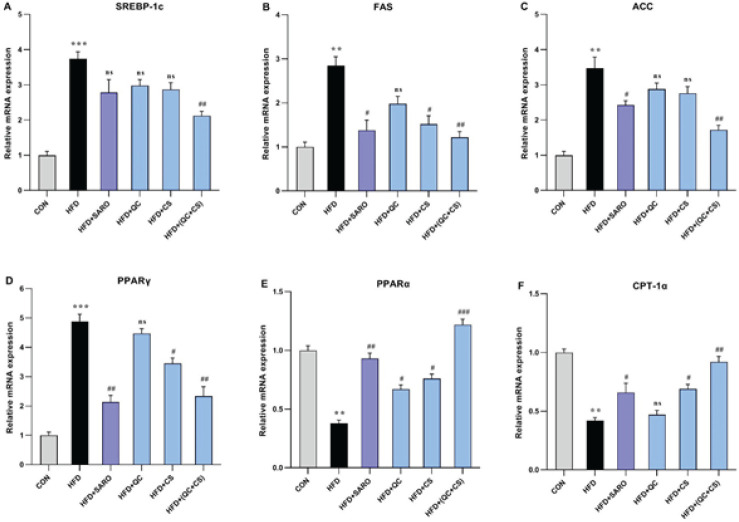
Assessment of the level of lipid metabolism genes in liver tissue of rats fed a high-fat diet (HFD)

In the combined CS+QC group, a noteworthy reduction was observed in hepatic mRNA expression levels of genes implicated in both lipid storage and mobilization. Specifically, the expression of Sterol Regulatory Element-Binding Protein 1c (SREBP-1c), Fatty Acid Synthase (FAS), Acetyl-CoA Carboxylase (ACC), and Peroxisome Proliferator-Activated Receptor Gamma (PPARγ) experienced a significant decrease. Simultaneously, there was an elevation in the expression levels of Peroxisome Proliferator-Activated Receptor Alpha (PPARα) and Carnitine Palmitoyltransferase I Alpha (CPT-1α)(Figure 3). Relative to the control group, the HFD group exhibited a substantial increase in the expression of SREBP-1c (Fold=3.74), FAS (Fold=2.85), ACC (Fold=3.45), and PPARγ (Fold=4.89) genes. However, the combined QC+CS group, as opposed to individual QC and CS groups, effectively attenuated this increase in gene expression (Figure 3 A, B, C, and D). Conversely, the HFD group displayed a reduction in the expression levels of PPARα (Fold=0.38) and CPT-1α (Fold=0.42) genes. Notably, the combined QC+CS treatment demonstrated a significant reversal of these effects (Figure 3 E, F).

***CS and QC combination reduces oxidative stress gene expression and ROS levels***

**Figure 4:**
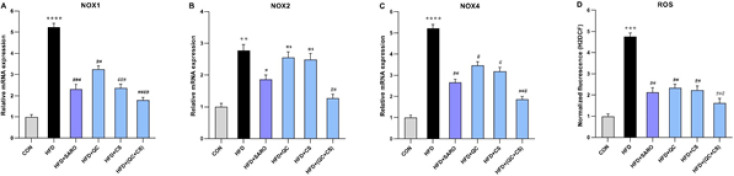
Evaluating the expression of genes involved in oxidative stress (A, B, and C) and ROS (D) production levels in liver tissues among different groups of Wistar rats

In comparison to the control group, there was a substantial increase in the expression of NOX1 (Fold=5.24), NOX2 (Fold=2.78), NOX4 (Fold=5.32) genes, and ROS levels (Fold=4.75) in the HFD group (Figure 4). Notably, the expression of the NOX1 gene witnessed a noteworthy reduction in the SARO, CS, QC, and QC+CS (Fold=1.79) groups (Figure 4A). Additionally, a significant decrease in NOX2 gene expression was observed in the SARO and QC+CS (Fold=1.25) groups, while no significant change was noted in the CS and QC groups (Figure 4B). The NOX4 gene expression exhibited a notable reduction in the SARO, CS, QC, and QC+CS (Fold=1.86) groups (Figure 4C). Similarly, the ROS level experienced a significant decrease in the SARO, CS, QC, and QC+CS (Fold=1.62) groups (Figure 4D).

***Combined CS and QC group modulates proinflammatory mRNA expression***

**Figure 5:**
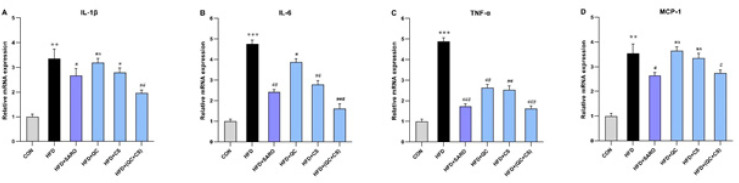
Assessment of the level of pro-inflammatory cytokines in liver tissue in the Wistar rats

In the combined CS+QC group, there was a significant reduction in the expression of proinflammatory genes, including IL-1β, IL-6, TNF-α, and MCP-1 (Figure 5). Relative to the control group, the HFD group exhibited a substantial increase in the expression of IL-1β (Fold=3.35), IL-6 (Fold=4.76), TNF-α (Fold=4.89), and MCP-1 (Fold=3.54) genes. However, the combined QC+CS group, as opposed to individual QC and CS groups, effectively attenuated this increase in gene expression (Figure 5 A, B, C, and D).

***Combined CS and QC group reduces fibrogenic genes mRNA expression***

**Figure 6:**
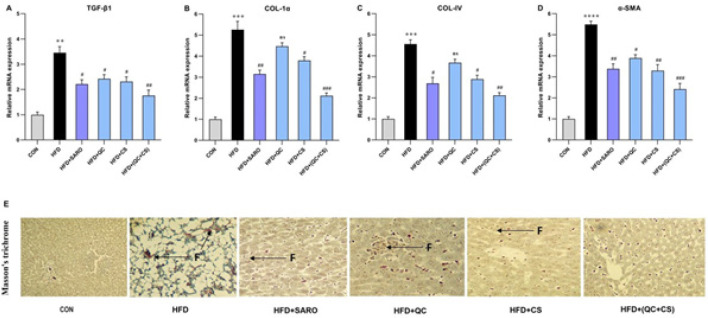
Effect of Saroglitazar (SARO), Capparis spinosa (CS), and Quercetin (QC) on the mRNA expression of α-SMA, Collagen1α (Col-1α), Collagen IV (Col-IV), and Transforming growth factor beta (TGF-β1) in activated HSCs

In the combined CS+QC group, there was a statistically significant decrease in the expression of fibrogenic genes such as Collagen (COL) and α-SMA (Figure 6). Relative to the control group, the HFD group exhibited a substantial increase in the expression of TGF-β1 (Fold = 3.46), COL-1α (Fold=5.25), COL-IV (Fold=4.56), and α-SMA (Fold=5.49) genes. However, the combined QC+CS group, as opposed to individual QC and CS groups, effectively attenuated this increase in gene expression (Figure 6 A, B, C, and D). In particular, TGF-β1 expression was diminished in the SARO, QC, and CS groups (Figure 6A). COL-1α expression saw a reduction in the SARO and CS groups, but not in the QC group (Figure 6B). A similar pattern was observed for COL-IV expression, which decreased in the SARO and CS groups but not in the QC group (Figure 6C). Lastly, α-SMA expression was lowered in the SARO, CS, and QC groups (Figure 6D). Using Masson’s trichrome staining, it was evident that the QC+CS treatment effectively attenuated the increased collagen deposition in the liver induced by HFD, as shown in Figure 6E.

***Combined CS and QC group suppresses the expression of the p-Smads proteins***

**Figure 7:**
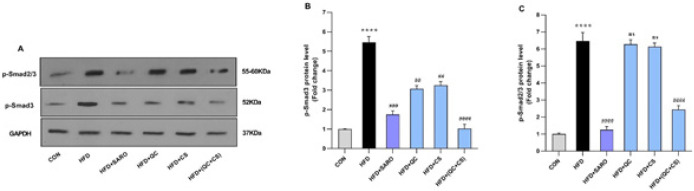
Level of protein expression in the p-Smad2/3 and p-Smad3 proteins in the Wistar rats

Relative to the control group, there was a substantial increase in the expression of p-smad3 (Fold=5.46) and p-smad2/3 (Fold=6.48) proteins in the HFD group (Figure 7). In the combined CS+QC treatment group, a statistically significant decrease was observed in the expression of p-smad2/3 (Fold=2.45) and p-smad3 (Fold=1.02) proteins. Notably, the SARO group exhibited decreased protein expression for p-smad3 and p-smad2/3. Similarly, decreases were noted in the QC and CS groups for p-smad3 expression, but not in p-smad2/3 expression (Figure 7A, B, and C).

**Table 1:** The macronutrient composition and caloric value of the high-fat mixture

High-fat-emulsion components	Amount
Corn oil (g)	400
Saccharose (g)	150
Total milk powder (g)	80
Cholesterol (g)	100
Sodium deoxycholate (g)	10
Tween 80 (g)	36.4
Propylene glycol (g)	31.1
Vitamin mixture (g)	2.5
Cooking salt (g)	10
Mineral mixture (g)	1.5
Distilled water (ml)	300
Total energy (kcal/l)	4342

**Table 2:** Primer pairs sequences

**Gene**	**Forward primer **	**Reversed primer**
SREBP-1c	TCTTGACCGACATCGAGACAT	CCTGTGTCTCCTGTCTCACC
FAS	CCCGGACCCAGAATACCAAG	TCTTCAAGTCACACGAGGTG
ACC	TTAAGGGGTGAAGAGGGTGC	CACTTCCAAAGACCTAGCC
PPARγ	CGAGTGTGACGACAAGGTGA	ACGCTTCTTCAATCTGTCTG
PPARα	TGGTGCATTTGGGCGTATCT	CACGAGCGCTAAGCTGTGA
CPT-1α	AGCCCTGAGACAGACTCACA	ATCACGAGGGTCCGTTTTCC
IL-1β	TGCCACCTTTGACAGTGATG	TGATGTGCTGCTGCGAGATT
IL-6	CCAGTTGCCTTCTTGGGACT	TGCCATTGCACAACTCTTTC
TNF-α	ATGGGCTCCCTCTCATCAGT	GCTTGGTGGTTTGCTACGAC
MCP-1	TGAGCACGTTTCAGTGAGCA	CCAACCTGCTTTACACCTAA
NOX1	AGGCTCCAGACCTCCATTGA	AAGGCAAGGCAGTTCCGAG
NOX2	GGCATTCGTAGTACAGCTCA	ATTGGTCCTCGGGAGTCAGA
NOX4	TGGCCAACGAAGGGGTTAAA	ACACAATCCTAGGCCAACA
TGF-β1	CTGCTGACCCCCACTGATAC	GGGGCTGATCCCGTTGATT
COL-1α	CCGATGGATTCCAGTTCGAGT	GGGACTTCTGAGGTTGCCA
COL-IV	GGCCCTTCATTAGCAGGTGT	GCTGGTGTGCATCACAAGG
α-SMA	GCCATCTTCATTGGGATGGA	CCCTGACAGGACGTTGTTA
GAPDH	CTCTCTGCTCCTCCCTGTTC	CGATACGGCCAAATCCGTTC

The amount of each gene was normalized to the amount of glyceraldehyde 3-phosphate dehydrogenase (GAPDH)

(A) Body weight in grams, (B) Body weight gain, (C) liver weight, and (D) liver TG were significantly increased in the High-fat diet (HFD) Rat model compared with the control group. After treatment with Saroglitazar (SARO), quercetin (QC), Capparis spinosa (CS), and QC+CS these variables decreased significantly compared to the HFD group. **P<0.01 and ***P<0.001 vs control group, and ^#^*P<*0.05, ^##^*P<*0.01, and ^###^*P<*0.001 vs HFD group. (E) Histopathological analysis of the HFD model before and after therapy with SARO, QC, CS, and a QC+CS combination. Typical images of 100X-magnified hematoxylin-eosin stained liver tissue from various treatment groups. (C: congestion; I: inflammation; S: steatosis)

SARO: Saroglitazar; QC: Quercetin; CS: Capparis spinosa

ALT, AST, and LDL-C levels show a significant increase, and HDL-C levels show a significant decrease in the High-fat diet group (HFD). These variables changed significantly in the drug-treated groups. SARO (Saroglitazar), QC (quercetin), and CS (Capparis spinosa) statistically significant in HFD versus the control group are shown with **P<0.01, *** *P<*0.001, and statistically significant in HFD versus other groups are shown with ^#^*P<*0.05; ^##^*P<*0.01; ^###^*P<*0.001

ALT: Alanine transaminase; AST: Aspartate transaminase; LDL-C: Low-density lipoprotein; HDL-C: High-density lipoprotein

Using quantitative real-time PCR, hepatic mRNA levels were measured and normalized to GAPDH mRNA expression. The values are shown as the mean standard deviation of fold changes compared to the CON (Control) group. ANOVA was followed by the Tukey-Kramer test for multiple comparisons to evaluate between-group differences. **P<0.01, ***P<0.001, and ****P<0.0001 vs control group, and, ^#^*P<*0.05, ^##^*P<*0.01, and ^###^*P<*0.001 vs HFD group

SREBP-1C, Sterol regulatory element-binding protein 1C; FAS, Farnesyl pyrophosphate synthase; ACC, Acetyl-CoA carboxylase; PPAR γ/α, peroxisome proliferator-activated receptor gamma /alpha and CPT-1 α, Carnitine Palmitoyltransferase I

Using quantitative real-time PCR, hepatic mRNA levels were evaluated and normalized to GAPDH mRNA expression. The values are given as the mean standard deviation of fold changes compared to the CON (Control) group. ANOVA, followed by the Tukey-Kramer multiple comparisons test, was used to examine between-group differences. **P<0.01, ***P<0.001, and ****P<0.0001 vs control group, and, ^#^*P<*0.05, ^##^*P<*0.01, ^###^*P<*0.001, and ^####^*P<*0.0001 vs HFD group

NOX1, 2, 4, NADPH oxidase1, 2, 4; ROS, Reactive oxygen species

GAPDH: Glyceraldehyde 3-phosphate dehydrogenase

Using quantitative real-time PCR, hepatic mRNA levels were evaluated and normalized to GAPDH mRNA expression. The values are given as the mean standard deviation of fold changes compared to the CON (Control) group. ANOVA, followed by the Tukey-Kramer multiple comparisons test, was used to examine between-group differences. **P<0.01 and ***P<0.001 vs control group, and, ^#^*P<*0.05, ^##^*P<*0.01, and ^###^*P<*0.001 vs HFD group

TNF- α: tumor necrosis factor-alpha; IL-6: interleukin 6; IL-1-β: interleukin 1-β; MCP-1: monocyte chemoattractant protein 1; GAPDH: Glyceraldehyde 3-phosphate dehydrogenase

The relative expression of TGF-β1 (A), Col-1α (B), Col-IV (C), and α-SMA (D) genes in HSCs activated with High-fat diet (HFD) increased significantly, and after treatment with SARO, QC, CS, and QC+CS, it was significantly reduced. The relative chain‐fold (2^−ΔΔCt^) of each gene was indicated as a column. **P<0.01, ***P<0.001, and ****P<0.0001 vs control group, and ^#^*P<*0.05, ^##^*P<*0.01, and ^###^*P<*0.001 vs HFD group. (E) Histopathological evaluation of the NASH model after treatment with SARO, QC, CS, and QC+CS. Images of liver tissue stained with Masson Trichrome at 100X magnification from different treatment groups. (F: Fibrosis)

(A) The effects of Capparis spinosa (CS) and Quercetin (QC) on the protein expression of p-Smad2/3 and p-Smad3. (B, C) Quantified plots of the impacts of CS and QC, individually and in combination, on the relative protein expression of p-Smad2/3 and p-Smad3, in High-fat diet (HFD) Rat model. (****P<0.0001, compared to untreated control; ^##^*P<*0.01 and ^###^*P<*0.001 and ^####^*P<*0.0001 compared to the HFD treated group). The GAPDH was used as the loading control

GAPDH: Glyceraldehyde 3-phosphate dehydrogenase

## Discussion

Steatohepatitis involves a complex process where fat accumulation in the liver is the initial event. *CS* contains many active chemical constituents, including flavonoids such as quercetin (QC)(24, 25). Our study explored CS+QC’s role in ameliorating NAFLD by regulating liver fat deposition via the TGF-β signaling pathway. In HFD rats, liver weight, body mass, and oxidative stress were significantly increased compared to control groups. The combined treatment of CS and QC reduced liver and blood lipids, liver weight, body weight gain, ROS, and inflammatory markers induced by HFD. This suggests that the combined intervention influences lipid metabolism and antioxidative and anti-inflammatory processes in the liver. Gene expression analysis also demonstrated that the combined treatment (CS+QC) could potentially reverse steatohepatitis. Our findings are consistent with those of Porras *et al*., (2017) who demonstrated that QC has a positive impact on lipid metabolism, and antioxidative and anti-inflammatory processes in the liver (26).

Acetyl-CoA carboxylase (ACC) regulates fatty acid oxidation and synthesis. QC may stimulate AMP-activated protein kinase (AMPK) to decrease ACC activity, promoting fat oxidation and reducing fatty acid synthesis. Peroxisome proliferator-activated receptors (PPARs) play crucial roles in modulating inflammation, lipid and glucose homeostasis, and fibrosis associated with NASH. The AMPK-PPAR axis is vital for lipid metabolism (27-29). Our investigation suggests that the interactive effect between QC and CS may activate the AMPK-PPAR axis, leading to decreased activity of SREBP-1c, FAS, ACC, and PPARγ, and increased activity of PPARα and CPT-1α. Our results align with those of another study (2015), which showed that QC reduces the activity of SREBP-1c, FAS, ACC, and the PPARγ signaling pathway (30).

A direct association exists between reactive oxygen species (ROS) and hepatic stellate cell (HSC) activation. ROS are primarily produced by NADPH oxidases (NOXs) and the cytochrome P450 (CYP450) family of enzymes. ROS participate in the TGF-β signaling system, facilitating a vicious cycle that promotes fibrosis. NAFLD has been linked to increased NOX1 expression, which impairs hepatic microcirculation (31-33). Our findings showed that ROS and the NOXs family were reduced in the combined treatment group (CS+QC), suggesting that QC protected liver cells from damage by decreasing oxidative stress and restoring antioxidant enzymes. Our findings are consistent with those of other researchers (2018), who demonstrated that QC decreases the activity of ROS and the NOXs family (34).

We investigated the role of PPARs in regulating crucial biological processes associated with NASH. PPARs primarily function to suppress inflammatory genes by inhibiting nuclear factor-kappa B (NF-κB) activity. TNF-α, an inflammatory cytokine, plays a significant role in the progression of NAFLD to NASH (5, 35). In our NASH model, we found that treatment with a PPAR agonist Saroglitazar (SARO) could reduce the increase of inflammatory cytokines and TNFα as a fibrotic marker, but the effects of the combination of QC and CS were stronger. In our NASH model, we discovered that treatment with a PPAR agonist (SARO) could reduce the increase of inflammatory cytokines and TNF-α as a fibrotic marker. However, the effects of the combined QC and CS treatment were more potent. This might be attributed to QC’s inhibition of inflammatory agents and TGF-β, which consequently reduces collagen 1 and 4 production and α-SMA, thereby inhibiting liver fibrosis. Our findings suggest a potential interactive effect between CS and QC, which may contribute to anti-inflammatory and anti-fibrotic effects. Our findings are consistent with those of a research (2011) that demonstrated that QC inhibits TGFβ, leading to a reduction in the production of collagen 1 and 4 (36).

In the current study, plasma indicators of NASH, ALT, and AST were reduced after six weeks of QC+CS therapy compared to SARO or QC alone. Our results also showed that QC+CS therapy stabilized body mass, liver weight, and liver indices while improving lipid profile, glycemic index, and weight reduction in rats with NAFLD. These findings align with clinical studies (37, 38) reporting that SARO, a dual agonist, decreased liver enzyme levels in individuals with NAFLD and diabetic dyslipidemia. 

The production of pro-inflammatory cytokines by Kupffer cells (KCs) played a significant role in the progression from NAFLD to NASH. TGF-β is a well-known growth factor released by KCs and is believed to be a key factor in inducing fibrosis (39-41). Our findings suggest that the interactive effect of QC and CS has the potential to attenuate systemic inflammation, possibly through QC’s influence on TGF-β. This influence may occur by inhibiting SMAD2/3 and SMAD3 phosphorylation in HFD rats, thereby inhibiting the TGF-β pathway and consequently reducing fibrosis. Additionally, our results indicate that QC affects TGF-βR1-SMAD2/3 signaling in lipid homeostasis, suggesting that TGF-βR1 may be a therapeutic target of QC. Our findings are consistent with studies (42) that report QC’s influence on TGF-β may be due to the inhibition of SMAD2/3 and SMAD3 phosphorylation.

## Conclusion

This study delved into the bioactive constituents of *C. spinosa* specifically, QC, and its effect on the molecular pathways that play a central role in the pathogenesis of NAFLD and NASH. Our biochemical analyses prove that *C. spinosa* compounds may exert regulatory influence over critical biological processes involved in these liver disorders, including inflammation, oxidative stress, lipid metabolism, and insulin sensitivity. Notably, our findings indicate a potential regulatory impact on TGF-β/SMADs signaling pathways, elucidating possible mechanistic pathways through which these phytochemicals may impart therapeutic benefit. However, it is essential to underscore that while our results indicate *C. spinosa* compounds’ influence on metabolic pathways, they do not directly provide evidence of attenuated necrosis within hepatic tissue. As such, while the modulation of the signaling pathways by *C. spinosa* and QC hints at a broader therapeutic efficacy, our study does not explicitly encompass the effects on hepatic necrosis. This distinction firmly positions the implications of our research within the precise parameters of the data obtained and calls for additional studies to thoroughly interrogate these compounds’ role in mitigating necrotic processes in liver diseases.

## Data Availability

The research data in the current study are available from the corresponding author upon reasonable request.

## References

[1] Younossi ZM, Loomba R, Rinella ME, Bugianesi E, Marchesini G, Neuschwander‐Tetri BA (2018). Current and future therapeutic regimens for nonalcoholic fatty liver disease and nonalcoholic steatohepatitis. Hepatology.

[2] Mota M, Banini BA, Cazanave SC, Sanyal AJ (2016). Molecular mechanisms of lipotoxicity and glucotoxicity in nonalcoholic fatty liver disease. Metabolism.

[3] Fang YL, Chen H, Wang CL, Liang LI (2018). Pathogenesis of non-alcoholic fatty liver disease in children and adolescence: From “two hit theory” to “multiple hit model”. World J Gastroenterol.

[4] Caligiuri A, Gentilini A, Marra F (2016). Molecular pathogenesis of NASH. Int J Mol Sci.

[5] Kakino S, Ohki T, Nakayama H, Yuan X, Otabe S, Hashinaga T (2018). Pivotal role of TNF-α in the development and progression of nonalcoholic fatty liver disease in a murine model. Horm Metab Res.

[6] Liarte S, Bernabé-García Á, Nicolás FJ (2020). Role of TGF-β in skin chronic wounds: A keratinocyte perspective. Cells.

[7] Pan X, Chiwanda Kaminga A, Liu A, Wen SW, Chen J, Luo J (2020). Chemokines in non-alcoholic fatty liver disease: A systematic review and network meta-analysis. Front Immunol.

[8] Brocker CN, Yue J, Kim D, Qu A, Bonzo JA, Gonzalez FJ (2017). Hepatocyte-specific PPARA expression exclusively promotes agonist-induced cell proliferation without influence from nonparenchymal cells. Am J Physiol Gastrointest Liver Physiol.

[9] Moseti D, Regassa A, Kim WK (2016). Molecular regulation of adipogenesis and potential anti-adipogenic bioactive molecules. Int J Mol Sci.

[10] Shiomi Y, Yamauchi T, Iwabu M, Okada-Iwabu M, Nakayama R, Orikawa Y (2015). A novel peroxisome proliferator-activated receptor (PPAR) α agonist and PPARγ antagonist, Z-551, ameliorates high-fat diet-induced obesity and metabolic disorders in mice. J Biol Chem.

[11] Eddouks M, Lemhadri A, Hebi M, Hidani AE, Zeggwagh NA, Bouhali BE (2017). Capparis spinosa L aqueous extract evokes antidiabetic effect in streptozotocin-induced diabetic mice. Avicenna J Phytomed.

[12] Ji YB, Yu L (2014). N-butanol extract of Capparis spinosa L induces apoptosis primarily through a mitochondrial pathway involving mPTP open, cytochrome C release and caspase activation. Asian Pac J Cancer Prev.

[13] Aichour R, Charef N, Baghiani A, Arrar L (2016). Immunomodulatory effects of algerian caper. Int J Pharm Pharm Sci.

[14] Miltonprabu S, Tomczyk M, Skalicka-Woźniak K, Rastrelli L, Daglia M, Nabavi SF (2017). Hepatoprotective effect of quercetin: From chemistry to medicine. Food Chem Toxicol.

[15] Hatami M, Kouchak M, Kheirollah A, Khorsandi L, Rashidi M (2023). Effective inhibition of breast cancer stem cell properties by quercetin-loaded solid lipid nanoparticles via reduction of Smad2/Smad3 phosphorylation and β-catenin signaling pathway in triple-negative breast cancer. Biochem Biophys Res Commun.

[16] Donaldson J, Ngema M, Nkomozepi P, Erlwanger K (2019). Quercetin administration post‐weaning attenuates high‐fructose, high‐cholesterol diet‐induced hepatic steatosis in growing, female, Sprague Dawley rat pups. J Sci Food Agric.

[17] Zou Y, Li J, Lu C, Wang J, Ge J, Huang Y (2006). High-fat emulsion-induced rat model of nonalcoholic steatohepatitis. Life Sci.

[18] Maciejewska D, Łukomska A, Dec K, Skonieczna-Żydecka K, Gutowska I, Skórka-Majewicz M (2019). Diet-induced rat model of gradual development of non-alcoholic fatty liver disease (NAFLD) with lipopolysaccharides (LPS) secretion. Diagnostics (Basel).

[19] Eddouks M, Lemhadri A, Michel JB (2004). Caraway and caper: Potential anti-hyperglycaemic plants in diabetic rats. J Ethnopharmacol.

[20] Akbari R, Behdarvand T, Afarin R, Yaghooti H, Jalali MT, Mohammadtaghvaei N (2021). Saroglitazar improved hepatic steatosis and fibrosis by modulating inflammatory cytokines and adiponectin in an animal model of non-alcoholic steatohepatitis. BMC Pharmacol Toxicol.

[21] Panchal SK, Poudyal H, Brown L (2012). Quercetin ameliorates cardiovascular, hepatic, and metabolic changes in diet-induced metabolic syndrome in rats. J Nutr.

[22] Kirsch R, Clarkson V, Shephard EG, Marais DA, Jaffer MA, Woodburne VE (2003). Rodent nutritional model of non‐alcoholic steatohepatitis: Species, strain and sex difference studies. J Gastroenterol Hepatol.

[23] Avni Y, Shirin H, Aeed H, Shahmurov M, Birkenfeld S, Bruck R (2004). Thioacetamide-induced hepatic damage in a rat nutritional model of steatohepatitis. Hepatol Res.

[24] Buzzetti E, Pinzani M, Tsochatzis EA (2016). The multiple-hit pathogenesis of non-alcoholic fatty liver disease (NAFLD). Metabolism.

[25] Eddouks M, Lemhadri A, Hebi M, Hidani AE, Zeggwagh NA, Bouhali BE (2017). Capparis spinosa L aqueous extract evokes antidiabetic effect in streptozotocin-induced diabetic mice. Avicenna J Phytomed.

[26] Porras D, Nistal E, Martínez-Flórez S, Pisonero-Vaquero S, Olcoz JL, Jover R (2017). Protective effect of quercetin on high-fat diet-induced non-alcoholic fatty liver disease in mice is mediated by modulating intestinal microbiota imbalance and related gut-liver axis activation. Free Radic Biol Med.

[27] Akbari R, Behdarvand T, Afarin R, Yaghooti H, Jalali MT, Mohammadtaghvaei NJBP (2021). Saroglitazar improved hepatic steatosis and fibrosis by modulating inflammatory cytokines and adiponectin in an animal model of non-alcoholic steatohepatitis. BMC Pharmacol Toxicol.

[28] Francque S, Szabo G, Abdelmalek MF, Byrne CD, Cusi K, Dufour JF (2021). Nonalcoholic steatohepatitis: The role of peroxisome proliferator-activated receptors. Nat Rev Gastroenterol Hepatol.

[29] Todisco S, Santarsiero A, Convertini P, De Stefano G, Gilio M, Iacobazzi V (2022). PPAR alpha as a metabolic modulator of the liver: Role in the pathogenesis of nonalcoholic steatohepatitis (NASH). Biology (Basel).

[30] Seo YS, Kang OH, Kim SB, Mun SH, Kang DH, Yang DW (2015). Quercetin prevents adipogenesis by regulation of transcriptional factors and lipases in OP9 cells. Int J Mol Med.

[31] Ma J, Li M, Kalavagunta PK, Li J, He Q, Zhang Y (2018). Protective effects of cichoric acid on H2O2-induced oxidative injury in hepatocytes and larval zebrafish models. Biomed Pharmacother.

[32] Hassan W, Rongyin G, Daoud A, Ding L, Wang L, Liu J (2014). Reduced oxidative stress contributes to the lipid lowering effects of isoquercitrin in free fatty acids induced hepatocytes. Oxid Med Cell Longev.

[33] Sancho P, Mainez J, Crosas-Molist E, Roncero C, Fernandez-Rodriguez CM, Pinedo F (2012). NADPH oxidase NOX4 mediates stellate cell activation and hepatocyte cell death during liver fibrosis development. PLoS One.

[34] Nambooppha B, Photichai K, Wongsawan K, Chuammitri P (2018). Quercetin manipulates the expression of genes involved in the reactive oxygen species (ROS) process in chicken heterophils. J Vet Med Sci.

[35] Gao H, Li Y, Chen X (2022). Interactions between nuclear receptors glucocorticoid receptor α and peroxisome proliferator–activated receptor α form a negative feedback loop. Rev Endocr Metab Disord.

[36] Nakamura T, Matsushima M, Hayashi Y, Shibasaki M, Imaizumi K, Hashimoto N (2011). Attenuation of transforming growth factor–β–stimulated collagen production in fibroblasts by quercetin-induced Heme oxygenase–1. Am J Respir Cell Mol Biol.

[37] Krishnappa M, Patil K, Parmar K, Trivedi P, Mody N, Shah C (2020). Effect of saroglitazar 2 mg and 4 mg on glycemic control, lipid profile and cardiovascular disease risk in patients with type 2 diabetes mellitus: a 56-week, randomized, double blind, phase 3 study (PRESS XII study). Cardiovasc Diabetol.

[38] Goyal O, Nohria S, Goyal P, Kaur J, Sharma S, Sood A (2020). Saroglitazar in patients with non-alcoholic fatty liver disease and diabetic dyslipidemia: A prospective, observational, real world study. Sci Rep.

[39] Stewart AG, Thomas B, Koff J (2018). TGF-β: Master regulator of inflammation and fibrosis. Respirology.

[40] Meng X-m, Nikolic-Paterson DJ, Lan HY (2016). TGF-β: The master regulator of fibrosis. Nat Rev Nephrol.

[41] Li H, Zheng HW, Chen H, Xing ZZ, You H, Cong M (2012). Hepatitis B virus particles preferably induce Kupffer cells to produce TGF-β1 over pro-inflammatory cytokines. Dig Liver Dis.

[42] Guo Y TY, Zhu H, Xiao Y, Guo H, Shang L, Zheng W (2021). Quercetin suppresses pancreatic ductal adenocarcinoma progression via inhibition of SHH and TGF-β/Smad signaling pathways. Cell Biol Toxicol.

